# Predicting Prolonged Length of ICU Stay through Machine Learning

**DOI:** 10.3390/diagnostics11122242

**Published:** 2021-11-30

**Authors:** Jingyi Wu, Yu Lin, Pengfei Li, Yonghua Hu, Luxia Zhang, Guilan Kong

**Affiliations:** 1 National Institute of Health Data Science, Peking University, Beijing 100191, China; joywu@pku.edu.cn (J.W.); zhanglx@bjmu.edu.cn (L.Z.); 2 Advanced Institute of Information Technology, Peking University, Hangzhou 311215, China; pfli@aiit.org.cn; 3 Department of Medicine and Therapeutics, LKS Institute of Health Science, The Chinese University of Hong Kong, Hong Kong, China; linyu@link.cuhk.edu.hk; 4 Department of Epidemiology and Biostatistics, School of Public Health, Peking University, Beijing 100191, China; yhhu@bjmu.edu.cn; 5 Medical Informatics Center, Peking University, Beijing 100191, China; 6 Renal Division, Department of Medicine, Peking University First Hospital, Peking University Institute of Nephrology, Beijing 100034, China

**Keywords:** prolonged length of ICU stay, machine learning, clinical decision rules, medical informatics

## Abstract

This study aimed to construct machine learning (ML) models for predicting prolonged length of stay (pLOS) in intensive care units (ICU) among general ICU patients. A multicenter database called eICU (Collaborative Research Database) was used for model derivation and internal validation, and the Medical Information Mart for Intensive Care (MIMIC) III database was used for external validation. We used four different ML methods (random forest, support vector machine, deep learning, and gradient boosting decision tree (GBDT)) to develop prediction models. The prediction performance of the four models were compared with the customized simplified acute physiology score (SAPS) II. The area under the receiver operation characteristic curve (AUROC), area under the precision-recall curve (AUPRC), estimated calibration index (ECI), and Brier score were used to measure performance. In internal validation, the GBDT model achieved the best overall performance (Brier score, 0.164), discrimination (AUROC, 0.742; AUPRC, 0.537), and calibration (ECI, 8.224). In external validation, the GBDT model also achieved the best overall performance (Brier score, 0.166), discrimination (AUROC, 0.747; AUPRC, 0.536), and calibration (ECI, 8.294). External validation showed that the calibration curve of the GBDT model was an optimal fit, and four ML models outperformed the customized SAPS II model. The GBDT-based pLOS-ICU prediction model had the best prediction performance among the five models on both internal and external datasets. Furthermore, it has the potential to assist ICU physicians to identify patients with pLOS-ICU risk and provide appropriate clinical interventions to improve patient outcomes.

## 1. Introduction

Intensive care units (ICU) provide complex and resource-intensive treatment for the sickest hospitalized patients. The need for critical care medicine has grown substantially over the past decade [1] and has consumed a huge portion of the income in many countries worldwide [2]. In the US, critical care medicine costs account for approximately 13% of hospital costs and 4% of national health expenditures [3]. Despite the huge investment in critical care medicine, medical resources in ICU are usually insufficient to meet the demands of ICU patients, especially in developing countries. Hospitals are under pressure to improve the efficiency and reduce costs for critical care. Length of stay in ICU (LOS-ICU) is a key indicator for medical efficiency [4] and critical care quality in hospitals [5]; a prolonged LOS-ICU (pLOS-ICU) generally leads to additional use of resources and thus increased medical costs [6,7]. A small percentage of patients with pLOS-ICU could consume a large proportion (nearly 50%) of ICU resource use [8,9]. The early identification of pLOS-ICU risk for ICU patients can provide not only an important reference for patient and family counseling but also an important indicator for optimal clinical interventions. However, ICU physicians can hardly accurately predict pLOS-ICU at ICU admission [10]. Effective pLOS-ICU prediction tools are strongly needed by the ICU physicians [4].

The present study sought to construct machine learning (ML) based models to perform pLOS-ICU prediction for general ICU patients. Four different ML methods, namely random forest (RF), support vector machine (SVM), deep learning (DL), and gradient boosting decision tree (GBDT), were used for prediction model development in this study. The reasons that we employed the above four ML methods are as follows. Firstly, SVM was a frequently used single ML method to deal with complex ICU data, and it showed robust performance in handling noisy and nonlinearly classified data [11,12]. Secondly, the ensemble learning method could combine multiple ML models to achieve better performance and generalizability than a single one [13], while RF and GBDT are typical ensemble learning models with different ensemble mechanisms. Thirdly, the emerging ML algorithm, DL, also showed good performance in supporting clinical decision making in ICU [14,15]. Therefore, the above four different ML methods were used for model development. Their prediction performance was compared with the customized SAPS II, which was used as benchmark model.

This article is structured as follows. Section 1 introduces the background and purpose of this study. Section 2 carries out a review of related work in the literature. Section 3 describes the datasets, study subjects, model development and evaluation methods used in this study. Section 4 shows various results generated in this study, and the results are then further discussed in Section 5. Finally the conclusions of this study are summarized in Section 6.

## 2. Related Work

In the literature, some pLOS-ICU prediction models have been developed. Among them, some were customized from traditional severity scoring systems, such as the simplified acute physiology score (SAPS) II [16], acute physiology and chronic health evaluation (APACHE) III score [17], and the sequential organ failure assessment (SOFA) score [18]. Alternatively, some were developed via the logistic regression (LR) method. For example, Zoller et al. [16] customized the SAPS II to predict pLOS-ICU based on a prospective single-center dataset and found that the customized SAPS II showed limited accuracy and utility. Houthooft et al. [18] customized the SOFA score to predict pLOS-ICU using a single-center dataset in Belgium, and the corresponding sensitivity was low. Herman et al. [6] used the LR method to develop a pLOS-ICU prediction model for patients undergoing isolated coronary artery bypass grafting (CABG), but the model was constructed using a single-center dataset with a small sample size. Rotar et al. [19] also developed a pLOS-ICU prediction model for patients following CABG surgery based on least absolute shrinkage and selection operator (Lasso) algorithm. Their model was internally tested with an area under the receiver operation characteristic curve (AUROC) of 0.72. However, no external validation was performed for all the above-mentioned pLOS-ICU prediction studies.

In recent years, ML algorithms have been used to develop prediction models in complex clinical contexts, such as ICU [20,21,22,23]. Meiring et al. [24] used four ML methods to predict ICU mortality and compared their prediction performance with APACHE-II and the traditional LR model. They found that all the four ML models outperformed APACHE-II and LR. Lin et al. [20] constructed three ML models, namely, artificial neural network, SVM, and RF, together with a customized SAPS II model, to predict the mortality of acute kidney injury patients in ICU. They found that RF model outperformed the three ML models, and all ML models performed much better than the customized SAPS II model. DL as an emerging ML algorithm in the past decade, and it has been broadly studied in healthcare studies to support clinical decision-making [14,15]. Viton et al. [25] utilized a DL model based on convolutional neural networks (CNN) to predict the risk of in hospital mortality using the real time series records in ICU, and their experimental results showed a competitive accuracy. Despite its accuracy, as a black-box model, DL is hard to explain and thus has difficulties in gaining clinicians’ acceptance. Qian et al. [13] performed comparison study of six ML models for early prediction of acute kidney injury in ICU, and their results showed that the GBDT model outperformed both LR and CNN models.

However, limited studies have used ML methods to develop models for predicting the pLOS-ICU among general ICU patients. Navaz et al. [26] developed a decision tree-based pLOS-ICU prediction model, and the results showed that the prediction performance of the decision tree model is poor. Rocheteau et al. [27] developed a DL model by combining temporal convolution and pointwise convolution to predict numeric LOS-ICU, but their model showed a limited performance with a R^2^ of 0.40 in external validation. Ma et al. [28] constructed a model to predict whether an ICU patient can be discharged after 10 days by combining just-in-time learning and one-class extreme learning, but the dataset used for model training and test was a small sample with only 4000 records and no external validation was conducted. A rough comparison of the related work in the literature with our proposed model is shown in Table 1.

The existing pLOS-ICU prediction models for general ICU patients are not effective enough as required by ICU clinicians [18,26,27,29]. In addition, most of the existing pLOS-ICU prediction models have not been externally validated [4,19,28,30], and the generalizability of these models is unknown. Therefore, more accurate prediction models with external validation are needed for pLOS-ICU prediction. A well-developed pLOS-ICU prediction model has the potential to assist ICU physicians to identify patients at a high risk of prolonged ICU stay, and thus may help improve clinical decision making and family counseling quality.

## 3. Materials and Methods

### 3.1. Datasets and Study Subjects

A publicly available critical care database, eICU Collaborative Research Database (eICU-CRD) [31], was used for model development and internal validation. The eICU-CRD database is a multicenter database that is maintained by the Laboratory for Computational Physiology (LCP) at the Massachusetts Institute of Technology (MIT), which has partnered with the eICU Research Institute. The database contains medical records of 200,859 admissions for 139,367 patients admitted to 335 units in 208 hospitals from 2014 to 2015 across the United States. Data include vital sign measurements, laboratory tests, care plan documentation, diagnosis information, treatment information, and others. All protected health information was deidentified, and no patient privacy data can be identified.

Another publicly available critical care database, the Medical Information Mart for Intensive Care (MIMIC) III [32], was used for external validation of the developed models. The MIMIC-III database, which is also maintained by LCP at MIT, is a single-center database. MIMIC-III contains 53,423 medical records of 38,597 adult patients admitted to critical care units at the Beth Israel Deaconess Medical Center (BIDMC) in Boston, Massachusetts between 2001 and 2012. MIMIC-III is also deidentified; variables and data types in MIMIC-III are similar to eICU-CRD. The source hospital of MIMIC-III does not participate in the eICU program. Thus, the MIMIC-III database is a completely independent dataset. A brief comparison of eICU-CRD and MIMIC-III is shown in Table 2.

**Figure 1:**
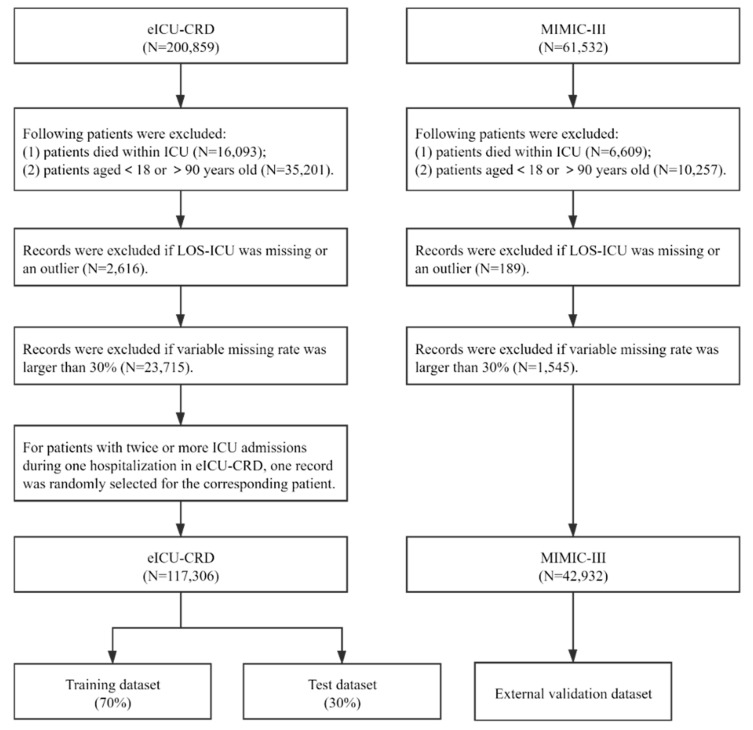
The procedure of study population selection.

In this study, all ICU records of patients between 18 and 90 years old were extracted from the eICU-CRD. Patients were excluded if they met the following criteria: variable missing rate was larger than 30%; or LOS-ICU was missing or an outlier, defined as a LOS-ICU above the 99th percentile of LOS-ICU in the studied dataset [33,34]. In addition, patients who died within ICU were also excluded, as the LOS-ICU pattern of patients who died within ICU may be different from the patients who survived in the ICU [7]. For patients with twice or more ICU admissions during one hospitalization in eICU-CRD, we randomly selected one record for the corresponding patient to ensure that all observations were independent in model development. Compared with the approach of selecting the first admission record for a patient having multiple ICU admissions during a hospitalization, randomly selecting one ICU record for the patient may help include patients with varying severities [35]. In MIMIC-III, patient data were extracted following the same inclusion and exclusion criteria. The difference is that we kept all the ICU records for patients with multiple ICU admissions during a hospitalization in MIMIC-III for external validation. The flowchart of the process for the patients’ inclusion is shown in Figure 1.

### 3.2. Primary Outcome and Predictor Variables

The primary outcome of this study was having pLOS-ICU. pLOS-ICU is defined as a length of stay longer than the reported average LOS-ICU [16,19,36], which is three days for general ICU patients in the United States [37].

To compare the prediction performance of four ML-based models with the customized SAPS II in an objective way, we used all predictor variables in the customized SAPS II to construct the SVM, RF, DL, and GBDT models. A total of 17 variables are used in the SAPS II scoring system, consisting of age, chronic diseases (metastatic cancer, acquired immunodeficiency syndrome, and hematologic malignancy), type of admission (scheduled surgical, unscheduled surgical, or medical), and 12 routine physiological measurements during the first 24 h in ICU [38]. The 12 physiological measurements include body temperature, heart rate, Pao_2_/Fio_2_ ratio, systolic blood pressure, urinary output, white blood cell count, serum urea nitrogen level, serum sodium level, serum potassium level, bilirubin level, serum bicarbonate level, and Glasgow Coma Score. Some of the physiological variables are time-stamped variables, which have multiple time-variant measurements. Similar to the original SAPS II [38], the time-stamped variables in the customized SAPS II, were scored on the basis of the worst value during the first 24 h, either minimum or maximum. In the SVM, RF, DL, and GBDT models, we used both the minimum and maximum values during the first 24 h of time-stamped variables as parallel inputs since the minimum and maximum values may reflect two different extreme physical conditions of one patient [20]. The chronic diseases were identified using International Classification of Diseases codes. As to the ICU admission type, its variable and corresponding options recorded in the two databases are different. By referring to the original SAPS-II [38], we used three different admission types: scheduled surgical, unscheduled surgical, and medical, to categorize the ICU admission type. In eICU-CRD, a patient would be identified as: (1) scheduled surgical if the ADMISSION_TYPE is ELECTIVE and he or she had surgery during the stay; (2) unscheduled surgical if the ADMISSION_TYPE is not ELECTIVE and he or she had surgery during the stay; and (3) medical if the ADMISSION_TYPE is none of the above. In MIMIC-III, a patient would be identified as: (1) scheduled surgical if variable electiveSurgery equals 1; (2) unscheduled surgical if variable electiveSurgery equals 0; and (3) medical if variable electiveSurgery is blank. The distributions of admission type in the eICU-CRD and MIMIC-III databases were similar.

### 3.3. Model Development

#### 3.3.1. Support Vector Machine (SVM)

SVM is a supervised ML algorithm that attempts to find an optimal separating hyperplane in the feature space for classification [39]. SVM has good prediction performance on either linearly or nonlinearly separable datasets, especially on the latter [40]. An SVM model can transform a nonlinearly separable dataset from the original feature space to a high-dimensional space and find a maximum-margin hyperplane to make classifications. Suppose a nonlinearly separable dataset *D* with *N* labeled cases is available; $D=\left\{\left(x_{1},\;y_{1}\right),\left(x_{2},\;y_{2}\right),\ldots ,\;\left(x_{i},y_{i}\right),\;\ldots ,\left(x_{N},y_{N}\right)\right\}$, where $y_{i}\in \left\{-1,\;1\right\}$. If ϕ(x) is the function for transforming dataset *D* to a high-dimensional space, then the classification hyperplane in the high-dimensional space can be defined if it satisfies the following equation:

(1) $$w^{T}\phi \left(x\right)+b=0,$$

where *w* and *b* are parameter vectors, *w* is a normal vector determining the direction, and *b* is the bias. The margin $r_{i}$ between a case $\left(x_{i},y_{i}\right)\;$and the hyperplane in the high-dimensional space is defined as follows:

(2) $$r_{i}=\frac{\left|w^{T}\phi \left(x_{i}\right)+b\right|}{\left\|w\right\|}.$$

The cases nearest to the hyperplane are called support vectors, which satisfy

(3) $$\left|w^{T}\phi \left(x\right)+b\right|=1.$$

The margin *R* between the support vector and the hyperplane is

(4) $$R=\frac{1}{\left\|w\right\|}.$$

The hyperplane that makes the margin *R* maximum is the optimal separating hyperplane (i.e., maximum-margin hyperplane). If we use $\hat{w}$ and $\hat{b}$ to denote the parameter vectors of the optimal hyperplane, then the optimal hyperplane can be expressed using the following equation:

(5) $${\hat{w}}^{T}\phi \left(x\right)+\hat{b}=0.$$

In the process of finding the optimal separating hyperplane, a kernel function is usually used to deal with the high computational cost. Commonly used kernel functions include the polynomial, linear, exponential and radial basis function kernels. A new instance $x_{new}$ is then classified by the trained SVM model with an optimal separating hyperplane as follows:

(6) $$y_{new}=\{\begin{array}{c} \;\;\;1,\;\;\;\;\;\;\;\;\;if\;f\left(x_{new}\right)>0\\ -1,\;\;\;\;\;\;\;\;\;if\;f\left(x_{new}\right)<0 \end{array}.\;$$

We used the sklearn.svm package in Python to construct the SVM model [41], and a set of optimal parameters of the SVM model were found using grid search, which is an exhaustive searching method that uses a manually specified subset of hyperparameter space to find the optimal parameters of a learning algorithm [42]. The SVM model obtained in this study had the following parameters: the kernel function was a radial basis function kernel. Gamma in the kernel function was 0.04, and the penalty parameter *C* was 1.

#### 3.3.2. Random Forest (RF)

RF is an ensemble learning model consisting of a multitude of decision trees [43]. Compared with single basic classifiers, ensemble learning models can combine the outputs of multiple basic classifiers and achieve an improved prediction performance [44]. An RF model is trained as follows: First, *K* (a tunable parameter, *K* = 100 in our study) subsets of a training dataset *D*, $\left\{D_{1},\;D_{2},\;\ldots ,\;D_{K}\right\}$ are generated using the bootstrap sampling method. The sampling proportion is $1-{\left(1-\frac{1}{N}\right)}^{N}$, where *N* is the total number of cases in the entire training dataset. Second, *K* decision trees $\left\{T_{1},\;T_{2},\;\ldots ,\;T_{K}\right\}$ are generated from the *K* subsets of training dataset separately. In decision tree induction, a total of *M* predictor variables is assumed, and *F* (a tunable parameter with *F* < *M*) out of *M* variables would be randomly selected for each node splitting based on the minimum impurity principle. Gini index is an indicator to measure information impurity, and it is frequently used in decision tree training [20]. For each tree, a variable or feature should not be used for node splitting any more if it has already been used for previous node splitting. For a dataset *D* containing samples with *J* classes, the Gini index of *D* is defined as follows [45]:

(7) $$\mathrm{Gini}\left(D\right)=1-\overset{J}{\underset{j=1}{\sum }}p_{j}^{2},$$

where $p_{j}$ is the frequency of the *j*th class in the dataset *D*. If a dataset *D* can be split into two subsets *D*^1^ and *D*^2^ by the variable *V*, then the decrease in Gini index *S* caused by this variable *V* is

(8) $$S\left(V\right)=\mathrm{Gini}\left(D\right)-\mathrm{Gini}\left(D^{1}\right)-\mathrm{Gini}\left(D^{2}\right).$$

The variable with a maximum decrease in Gini index is then used for node splitting in a decision tree growth. After all the *K* trees have been generated, an RF model forms. In a RF-based inference or classification, the clinical data of a new patient are inputs to the model, the outputs of all the *K* decision trees are aggregated through a voting algorithm, and then the majority vote is declared as the final classification.

RF model training has two types of randomizations: randomization of training datasets and randomization of feature subsets in its basic decision tree growth. This classification helps reduce the scale and dimension of the training dataset in generating decision trees. These two randomizations enable an RF model to deal with high-dimensional and large-scale data.

We used the sklearn.RandomForestClassifier package in Python to construct the RF model in this study [41]. A set of optimal parameters of the RF model were found using grid search. The RF model obtained in this study had the following parameters: the number of decision trees *K* was 100; the number of variables selected for each node splitting *F* was the square root of the number of input variables, sqrt(*M*); and the minimum number of samples required to split an internal node was 2.

#### 3.3.3. Gradient Boosting Decision Tree (GBDT)

GBDT is also a kind of ensemble learning model that uses decision trees as the basic classifier [46]. In contrast to parallel decision trees in an RF model, decision trees in a GBDT model are serially generated. A decision tree in a GBDT model is trained based on the bias of all the previous decision trees in the model. In its inference process, a GBDT model synthesizes outputs of the serial decision trees through an addition algorithm to make classifications.

The training of a GBDT model proceeds as follows: suppose a training dataset *D* with *N* labeled cases is available; $D=\left\{\left(x_{1},\;y_{1}\right),\left(x_{2},\;y_{2}\right),\ldots ,\;\left(x_{i},y_{i}\right),\;\ldots ,\left(x_{N},y_{N}\right)\right\}$, where $y_{i}\in \left\{-1,\;1\right\}$. The decision trees are generated iteratively in a GBDT model training, and each tree in the model is trained based on the bias between the observed outcomes and the predicted probabilities generated by all its previous trees. Therefore, we need to set an initial predicted probability $f_{0}\left(x_{i}\right)$ for a training case $\left(x_{i},y_{i}\right)$ before the generation of the first decision tree in a GBDT model. The initial predicted probability of the case $\left(x_{i},y_{i}\right)$ is defined as

(9) $$f_{0}\left(x_{i}\right)=\frac{1}{2}ln\frac{P\left(y=1|x\right)}{P(y=-1|x)},$$

where P(y=1|x) is the frequency of class y=1 in the dataset *D*, and P(y=−1|x) is the frequency of class y=−1 in the dataset *D*. The bias between the probability $f_{0}\left(x_{i}\right)$ and $y_{i}$, i.e., the observed outcome of $x_{i}$, is defined as residual $r_{0,i}$, which is calculated using the following equation:

(10) $$r_{0,i}=\frac{2y_{i}}{1+exp\left(2y_{i}f_{0}\left(x_{i}\right)\right)}\;.$$

In the first round of iteration, the training dataset for the first tree training is constructed as $D_{1}=\left\{\left(x_{1},\;r_{0,1}\right),\left(x_{2},\;r_{0,2}\right),\ldots ,\;\left(x_{i},r_{0,i}\right),\;\ldots ,\left(x_{N},r_{0,N}\right)\right\}$ and a decision tree stops growing when no more decrease occurs in its prediction error or it has achieved a preset threshold of max depth. Then, the generated decision tree can be used to predict the bias between the initial probability and the actual outcome for all cases and obtain results $T_{1}=\left\{t_{1,1},\;t_{1,2},\;\ldots ,t_{1,i},\;\ldots ,\;t_{1,N}\right\}$ Afterward, the predicted probability for $x_{i}$, $f_{1}\left(x_{i}\right)$ based on the first decision tree can be calculated as

(11) $$f_{1}\left(x_{i}\right)=f_{0}\left(x_{i}\right)+t_{1,i}\;.$$

After prediction results have been generated using the first decision tree, a second residual $r_{1,i}$ between the predicted probability and the actual outcome can be generated, and then a second training dataset $D_{2}=\left\{\left(x_{1},\;r_{1,1}\right),\left(x_{2},\;r_{1,2}\right),\ldots ,\;\left(x_{i},r_{1,i}\right),\;\ldots ,\left(x_{N},r_{1,N}\right)\right\}$ can be constructed for the second decision tree training.

Similarly, in the *k*th round of iteration, the *k*th (k=1, 2, … , K; *K* is the total number of decision trees and is a tunable parameter; *K* = 100 in our study) decision tree is generated based on the bias of the previous (*k* − 1) decision trees.

(12) $$r_{k-1,i}=\frac{2y_{i}}{1+exp\left(2y_{i}f_{k-1}\left(x_{i}\right)\right)}.$$

Then, the training dataset for the *k*th tree $D_{k}=\left\{\left(x_{1},\;r_{k-1,1}\right),\left(x_{2},\;r_{k-1,2}\right),\ldots ,\;\left(x_{i},r_{k-1,i}\right),\;\ldots ,\left(x_{N},r_{k-1,N}\right)\right\}$ can be constructed. The *k*th generated tree predicts the bias for all cases and obtains $T_{k}=\left\{t_{k,1},\;t_{k,2},\;\ldots ,t_{k,i},\;\ldots ,\;t_{k,N}\right\}$. The predicted probability for $\left(x_{i},y_{i}\right)$ after the *k*th round of iteration $f_{k}\left(x_{i}\right)$ is calculated as

(13) $$f_{k}\left(x_{i}\right)=f_{k-1}\left(x_{i}\right)+t_{k,i}.$$

After all the *K* decision trees have been generated using a forward stage-wise algorithm, a GBDT model forms. For a trained GBDT model, when a new instance $x_{new}$ is inputted into the model, each tree makes a prediction about the bias between the actual outcome and the predicted probability, given by all its previous trees for the new instance, and obtains $\left\{t_{1,new},\;t_{2,new},\;\ldots ,\;t_{k,new},\;\ldots ,\;t_{K,new}\;\right\}$. Then, the model combines the predictions of all *K* decision trees using an addition algorithm, as follows:

(14) $$F\left(x_{new}\right)=f_{0}\left(x\right)+\overset{K}{\underset{k=1}{\sum }}t_{k,new}.$$

The final predicted probability for $x_{new}$ generated by the GBDT model is

(15) $$P_{new}=\frac{1}{1+exp\left(-2F\left(x_{new}\right)\right)}.$$

In addition, a GBDT model can provide a variable importance ranking based on the variable importance weight generated by all its basic decision trees.

We used the sklearn.GradientBoostingClassifier package in Python to construct the GBDT model in this study [41]. A set of optimal parameters of the GBDT model were found using grid search. The GBDT model obtained in this study had the following parameters: the fraction of samples used for each decision tree was 0.7, the number of decision trees *K* was 100, the number of variables selected for each node splitting was the square root of the number of input variables, the maximal depth of each decision tree was 6, and the minimum number of samples required to split an internal node was 200.

#### 3.3.4. Deep Learning (DL)

DL models [47] were developed from artificial neural networks. A DL model was constructed with a greedy layer-by-layer method, where multiple layers were used to progressively extract higher-level features from the raw input and pick out features with high predictive value. DL models have been broadly used in healthcare studies to support clinical decision-making, such as diagnosis [48], prognosis prediction [49], and resource allocation [50].

General architecture of a DL model consists of an input layer, multiple hidden layers, and an output layer. Each layer contains a set of neurons, and is fully connected with its adjacent layers. A neuron receives a signal, processes it, and then signals neurons connected to it. Signals travel from the first (input), to the last (output) layer. In the input layer, the number of neurons was determined by the number of input features. Each neuron in the input layer represents an input feature. In the hidden layer, neurons transform the signals from the input layer (or the previous hidden layer) with a weighted summation followed by a non-linear activation function. Starting from initial random weights, the weights between neurons were repeatedly updated using optimization algorithm to minimize the loss function. The process of weight training stops when it reaches a preset maximum number of iterations, or when the improvement in loss is below a certain number. At last, the output layer receives values from the last hidden layer and transforms them into outcome values.

In this study, a multilayer perceptron algorithm (MLP) was employed to construct a DL model. MLP is the most typical DL algorithm. Compared to complex DL architecture such as CNN, MLP has a relatively small number of parameters and is less complex. Prediction models based on MLP is expected to be more acceptable in clinical practice than CNN [51]. The sklearn.MLPClassifier package in Python was used for model development [41]. A set of optimal parameters of the DL model were found using grid search. The DL model obtained in this study had the following parameters: the optimization algorithm was Limited-memory BFGS (L-BFGS); the number of hidden layers was 2, the number of neurons in each hidden layer was (100, 100); and the penalty parameter was 10.

#### 3.3.5. Customized SAPS II

The SAPS II model is a commonly used scoring tool in ICU to assess the severity of illness, and it is frequently used as a benchmark model for performance comparison in prognosis prediction model development [20,52,53]. SAPS II was developed by LeGall et al. [38] in 1993 on the basis of the clinical dataset of 12,997 ICU patients. As the original SAPS II tool was developed from a clinical dataset of 1993 via traditional logistic regression, clinicians and researchers usually use newly collected dataset to customize the coefficients for target population [54,55]. In the literature, some studies have carried out the work of customizing SAPS II for pLOS-ICU prediction [16,18]. By referring to those studies, we developed a customized SAPS II model for pLOS-ICU prediction using the eICU-CRD database, and then used the customized SAPS II as the benchmark to evaluate the prediction performance of ML models developed in this study.

The algorithm behind the SAPS II model is the LR algorithm. A total of 17 predictor variables are used in the SAPS II scoring system, and each variable is assigned a different score between 0 and 26 according to each patient’s condition. The coefficient of each variable obtained from a multivariate LR analysis, is used as a criterion for assigning a score to the variable. Then, the assigned score can be used to rank variable importance. In SAPS II, the in-hospital mortality probability of an ICU patient can be calculated based on the overall score using the following formula:

(16) $$z=\beta_{0}+\beta_{1}\times Score+\beta_{2}\times \ln\left(Score+1\right),$$

(17) $$P_{mor}=\frac{1}{1+e^{-z}},$$

where *Score* is the overall SAPS II score of a specific ICU patient; $\beta_{0}$, $\beta_{1}$, and $\beta_{2}$ are the coefficients generated via the LR algorithm; and $P_{mor}$ is the mortality probability of the ICU patient.

We developed a customized SAPS II model for pLOS-ICU prediction using the eICU-CRD database. In model customization, the coefficients $\beta_{0}$, $\beta_{1}$, and $\beta_{2}$ were re-estimated based on the eICU-CRD training dataset, and a new set of coefficients $\beta \prime_{0},\;\beta \prime_{1},\;\mathrm{and}\;\beta \prime_{2}$ were generated. In our customized SAPS II model, the probability of pLOS-ICU for a patient $P_{pLOS-ICU}$ can be calculated as follows:

(18) $$z_{new}=\beta \prime_{0}+\beta \prime_{1}\times Score+{\beta^{\prime }}_{2}\times \ln\left(Score+1\right),$$

(19) $$P_{ICU-LOS}=\frac{1}{1+e^{-z_{new}}}.$$

The customized SAPS II model used in this study is as follows.

(20) $$z_{new}=-1.88+0.06\times Score-0.27\times \ln\left(Score+1\right),$$

(21) $$P_{ICU-LOS}=\frac{1}{1+e^{-z_{new}}}.$$

### 3.4. Model Validation

We randomly split the eICU-CRD dataset into two parts: 70% as training dataset and 30% as test dataset for internal validation. The entire MIMIC-III dataset was used for external validation. In model training, we used five-fold cross validation to find optimal parameters for the four ML-based models.

### 3.5. Analysis

We used PostgreSQL 10.5 (The PostgreSQL Global Development Group, Berkeley, California, United States) to extract data from the eICU-CRD and MIMIC-III databases. In the two extracted datasets, the missing value of each predictor variable was filled up using the median value after excluding unqualified patient records. Descriptive data are presented either as mean ± standard deviation or actual numbers (percentages). The prediction performance of the five models were measured using AUROC [56], area under the precision-recall curve (AUPRC) [25], estimated calibration index (ECI) [57], and Brier score [56]. AUROC measures the discrimination power of a prediction model, representing the ability of distinguishing between the positive and negative samples. A high AUROC value represents a strong discrimination power. AUPRC also measures the discrimination power of a model, while AUPRC pays more attention to the ability of identifying positive samples. Compared with AUROC, AUPRC is more sensitive to data imbalance. It should be noted that the baseline value of AUPRC for a model is equal to the fraction of positives in a classification task [58]. This means that the higher the AUPRC is (compared to the fraction of positives), the better performance a model can achieve. In this study, the fraction of positives (pLOS-ICU) is around 25%, and thus the baseline AUPRC is 0.25. Therefore, obtaining an AUPRC of more than 0.50 means a good pLOS-ICU prediction. ECI measures the calibration power of a model, representing the average difference between the predicted probability and the observed probability of each ICU patient. A low ECI suggests a strong calibration power. The Brier score is an overall performance measure, and a low Brier score suggests a superior overall performance. Another issue worth mentioning here is that the training dataset is imbalanced as patients with pLOS-ICU are the minority in eICU-CRD. To remove the effect that an imbalanced dataset may have on trained prediction models, a comprehensive performance measure considering both sensitivity and specificity instead of just prediction accuracy should be used as criteria for model evaluation. The AUROC value meets this need. A calibration plot was used to illustrate the calibration power of a model visually. The ideal calibration curve for a perfect model is a diagonal, which indicates that the predicted probabilities are consistent with the observed probabilities. The model with the best prediction performance was used to generate variable importance ranking, and the top five important predictor variables were presented.

## 4. Results

Overall, 117,306 ICU patients in eICU-CRD and 42,932 ICU patients in MIMIC-III were included for model derivation and validation. The characteristics of the ICU patients were similar in both databases (Table 3). The proportion of ICU patients with pLOS-ICU was 26.7% in eICU-CRD and 34.8% in MIMIC-III. In eICU-CRD, the proportion of male patients was 54.8%, and the age of all ICU patients was 61.6 ± 16.6 years. In MIMIC-III, the proportion of male patients was 57.6% and the age of all ICU patients was 62.0 ± 16.5 years.

The prediction performance of the five models on the internal and external validation datasets are compared in Table 4. On eICU-CRD (internal validation dataset), the GBDT model achieved the best overall performance (Brier score, 0.164), discrimination (AUROC, 0.742; AUPRC, 0.537), and second-best calibration (ECI, 8.224). On MIMIC-III, the external validation dataset, the GBDT model also achieved the best overall performance (Brier score, 0.166), discrimination (AUROC, 0.747; AUPRC, 0.536), and calibration (ECI, 8.294). The prediction performance of all the five models on eICU-CRD (internal validation dataset) was superior to that on MIMIC-III. On the internal dataset, the RF and GBDT models performed better than the customized SAPS II, but the SVM model performed slightly worse than the customized SAPS II in Brier score and ECI though it had better discrimination. Meanwhile, all the four ML models performed better than customized SAPS II on the external validation dataset.

**Figure 2:**
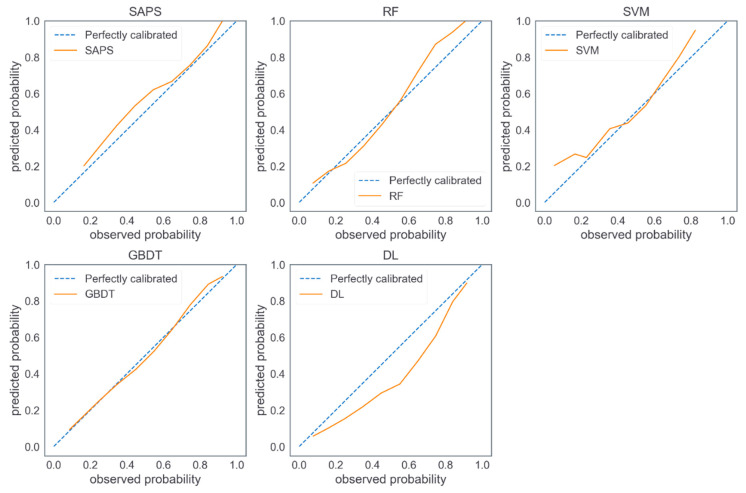
Calibration plots of the five models on MIMIC-III.

Figure 2 shows the calibration plots of the five models on MIMIC-III (external validation dataset). The calibration curve of the GBDT model was an optimal fitting. The customized SAPS II and SVM model tended to overestimate probabilities of pLOS-ICU in most ICU patients, whereas the DL model tended to underestimate probabilities of pLOS in most ICU patients, the RF model tended to underestimate the probabilities of pLOS-ICU in low-risk patients and overestimate the probabilities of pLOS-ICU in high-risk patients.

Top five predictive variables identified by GBDT and SAPS II models are listed in Table 5. Three variables, namely Glasgow Coma Score, systolic blood pressure, and white blood cell count, were ranked among top five important variables by both models.

## 5. Discussion

In this study, we used four ML methods, namely, SVM, RF, DL, and GBDT, to construct pLOS-ICU prediction models on the basis of eICU-CRD. Furthermore, we used MIMIC-III to validate the developed models externally. The four ML-based models were compared with the customized SAPS II, which is based on the traditional LR algorithm. The comparison results showed that the GBDT outperformed the other four models in terms of discrimination, calibration, and overall performance in either internal or external validation. The main contribution of this study was an optimal data-driven ML model for predicting pLOS-ICU risk, and the model had the following characteristics. First, although some pLOS-ICU prediction models have been developed in the literature [6,16,26], the GBDT-based model developed in this study showed better prediction performance than the state-of-the-art pLOS-ICU prediction models for general ICU patients. Second, most published pLOS-ICU prediction models have not been externally validated, while the GBDT-based prediction model developed in this study was externally validated and the validation results showed a satisfied prediction performance. Third, if clinical application is taken into consideration, a pragmatic pLOS-ICU prediction model could help physicians identify patients at high risk and thus may provide timely individualized interventions, and finally, patients’ prognosis may be improved. Therefore, from application perspective, the pLOS-ICU prediction model developed in this study is an innovational tool though it has limited contributions from the perspective of the ML method.

The RF model has the second-best prediction performance on both datasets. The good prediction performance of the GBDT and RF models may be due to the fact that both models are ensemble learning models, which make predictions by combining the outputs of corresponding basic classifiers, and thus can help reduce the bias that occurs in a single classifier. Our results also verified that ensemble models are usually superior to single models [44,59,60]. The GBDT model is slightly superior to the RF model in terms of all performance measures, probably since the basic decision trees in the two models were trained by two different approaches. In an RF model, decision trees are grown in a parallel way. In a GBDT model, trees are trained iteratively, and each decision tree is trained to correct the discrepancy of all its preceding decision trees. Thus, it helps generate a growing forest with decreasing prediction errors. Our results are consistent with previous studies [61,62,63], which indicated that the GBDT models outperform RF models in terms of prediction. All the four ML models yield superior performance over the customized SAPS II in external validation, although the performance of the customized SAPS II and SVM models slightly differ in internal validation. The possible reason is that ML algorithms have better generalizability compared with traditional LR models [64,65].

Application of ML techniques in healthcare has led to an increased emphasis for ML explainability. For most ML systems, an improved predictive accuracy may often be achieved through increased model complexity [66]. The prime example is the DL paradigm. However, explainability is highly associated with acceptance and promotability of a ML system in clinical practice. There is usually a trade-off between performance and explainability of a ML model. Compared to DL or SVM, GBDT based pLOS-ICU prediction models are less complex and more explainable. The decision trees in the GBDT model can be transformed to understandable decision rules, and thus GBDT may be more acceptable for clinicians. In addition, GBDT has the mechanism to rank the importance of predictable variables from a population perspective, and the importance ranking is intuitionistic for physicians to understand the association of clinical signs and symptoms with pLOS-ICU risk. However, explainability of GBDT is still limited in terms of individual prediction as it is hard to identify predictable variables for each individual prediction. In the literature, some studies have been carried out to improve ML explainability. For example, Ribeiro et al. [67] proposed a local interpretable model-agnostic explanations (LIME) method to generate explainability for individual prediction given by any black-box model. In LIME, an explainable model (such as a decision tree) can be developed based on neighboring instances (identified by the black-box model) of an individual instance. Lundberg et al. [68] proposed a Shapley additive explanations (SHAP) method to enhance explainability by computing the importance value of each feature based on the average marginal contribution to individual predictions. Therefore, ML explainability can be taken into consideration in future pLOS-ICU prediction model development.

The overall prediction performance of all five models on eICU-CRD (internal validation dataset) is superior to that of MIMIC-III. This result suggests that a possible degradation of prediction performance occurs when the models are applied to a new dataset. The internal and external validation datasets exhibit a slight difference in performance in our study, demonstrating a strong generalizability of the developed pLOS-ICU prediction models. Such strong generalizability may be attributed to the fact that the pLOS-ICU models were trained on the basis of a multicenter dataset, which is more population representative than a single-center dataset.

The top five important variables identified by the GBDT model include Pao2/Fio2 ratio, Glasgow Coma Score, serum urea nitrogen level, systolic blood pressure, and white blood cell count. Three variables (i.e., Glasgow Coma Score, systolic blood pressure, and white blood cell count) are also ranked among the top five by the SAPS II model. The Glasgow Coma Score is used to assess the level of consciousness in patients [69], and patients with decreased levels of consciousness tend to have poor prognosis [70,71]. The independent capabilities of systolic blood pressure and white blood cell count in predicting the prognosis of ICU patients have also been verified by existing studies [72,73]. The top five important variables identified by the two models only show a slight difference. As identified by the GBDT model, Pao2/Fio2 ratio and serum urea nitrogen level, in addition to Glasgow Coma Score, systolic blood pressure, and white blood cell count, may have potential influence on the prognosis of ICU patients. This finding may provide a clue for future research. Studies focusing on the detailed association between the prognosis of ICU patients and the Pao2/Fio2 ratio or serum urea nitrogen level are limited [74,75,76].

This study has several strengths. First, the database used to derive the prediction models is a large, multicenter database with a relatively representative population. Second, a large, single-center database (i.e., MIMIC-III) was used for external validation and helped assess the generalizability of the developed prediction models. Third, all the predictor variables used to construct prediction models are routinely collected during the first 24 h in ICU, thereby ensuring the feasibility of applying the prediction models in clinical practice to assist physicians in decision making.

However, this study has limitations. First, the eICU-CRD database contains only data of ICU patients admitted between 2014 and 2015 in the US, and the MIMIC-III dataset contains only data of ICU patients admitted to the BIDMC from 2001 to 2012. No data from other countries were used for model validation. Therefore, the clinical utility of the pLOS-ICU prediction models needs further assessment before application in other regions. Second, selection bias may exist since we excluded patients who died in the ICU. Accordingly, the pLOS-ICU prediction models developed in this study may not apply to patients who die in ICU. Third, to compare the prediction performance of four ML-based models with the customized SAPS II in an objective manner, we only included the predictor variables used in the customized SAPS II for model training. Other potential predictor variables may have been neglected in our study.

## 6. Conclusions

In summary, this study demonstrates that the GBDT model outperforms the other four developed models in pLOS-ICU prediction. As all the predictor variables can be available during the first 24 h in ICU, the GBDT-based pLOS-ICU prediction model has potential to assist ICU physicians in identifying patients with pLOS-ICU risk and thus make optimal clinical intervention decisions. This study lays a foundation for the future application of a GBDT-based pLOS-ICU prediction model in ICU clinical practice.

## Figures and Tables

**Table 1:** Characteristics of related work.

Category	Study	Population	Sample Size	Dataset	Outcome	Models	External Validation	Performance
Traditional regression based pLOS-ICU prediction models	Zoller et al. [16]	General ICU patients	110	Single-center	pLOS-ICU	Customized SAPS II	×	AUROC: 0.70
	Houthooft et al. [18]	General ICU patients	14,480	Single-center	pLOS-ICU	Customized SOFA score	×	Sensitivity: 0.71
	Herman et al. [6]	Patients undergoing CABG	3483	Single-center	pLOS-ICU	LR	×	AUROC: 0.78
	Rotar et al. [19]	Patients following CABG	3283	Single-center	pLOS-ICU	LASSO	×	AUROC: 0.72
ML-based models in ICU	Meiring et al. [24]	General ICU patients	22,514	Multicenter	ICU mortality	AdaBoost, RF, SVM, DL, LR, and customized APACHE-II	×	AUROC: 0.88 (DL)
	Lin et al. [20]	Acute kidney injury patients	19,044	Single-center	ICU mortality	ANN, SVM, RF, and customized SAPS II	×	AUROC: 0.87 (RF)
	Viton et al. [25]	General ICU patients	13,000	Single-center	ICU mortality	DL	×	AUROC: 0.85
	Qian et al. [13]	General ICU patients	17,205	Single-center	Acute kidney injury	XGBoost, RF, SVM, GBDT, DL, and LR	×	AUROC: 0.91 (GBDT)
ML-based pLOS-ICU prediction models	Navaz et al. [26]	General ICU patients	40,426	Single-center	pLOS-ICU	Decision tree	×	Accuracy: 0.59
	Rocheteau et al. [27]	General ICU patients	168,577	Multicenter	LOS-ICU	DL	√	R^2^: 0.40
	Ma et al. [28]	General ICU patients	4000	Single-center	pLOS-ICU	Combining just-in-time learning and one-class extreme learning	×	AUROC: 0.85
	Our study	General ICU patients	160,238	Multicenter	pLOS-ICU	RF, SVM, DL, GBDT, and customized SAPS II	√	-

**Table 2:** Characteristics of eICU-CRD and MIMIC-III.

Items	eICU-CRD	MIMIC-III
Country	United States	United States
Data	Multicenter	Single-center
Year	2014–2015	2001–2012
Number of units	335	1
Number of hospitals	208	1
Number of patients	139,367	38,597
Number of admissions	200,859	53,423
Deidentification	All protected health information was deidentified, and no patient privacy data can be identified.	
Data content	Vital sign measurements, laboratory tests, care plan documentation, diagnosis information, treatment information, and others.	

**Table 3:** Characteristics of ICU patients in eICU-CRD and MIMIC-III.

Items	eICU-CRD	MIMIC-III
Total number	117,306	42,932
Age/years	61.6 ± 16.6	62.0 ± 16.5
Gender, n (%)		
Male	64,244 (54.8%)	24,740 (57.6%)
Female	53,049 (45.2%)	18,192 (42.4%)
SAPS II score	30.0 ± 13.3	32.7 ± 12.7
LOS-ICU (IQR^1^)/day	1.8 (1.0–3.2)	2.1 (1.2–4.0)
PLOS-ICU, *n* (%)	31,296 (26.7%)	14,951 (34.8%)

IQR^1^, interquartile range.

**Table 4:** Prediction performance of the five models on eICU-CRD (internal) and MIMIC-III (external).

Models	eICU-CRD				MIMIC-III			
	Brier Score	AUROC	AUPRC	ECI	Brier Score	AUROC	AUPRC	ECI
Customized SAPS II	0.181	0.667	0.439	9.028	0.175	0.669	0.402	8.742
RF	0.166	0.735	0.530	8.317	0.169	0.745	0.530	8.469
SVM	0.183	0.690	0.480	9.137	0.172	0.716	0.482	8.577
DL	0.164	0.742	0.536	8.223	0.171	0.743	0.527	8.551
GBDT	0.164	0.742	0.537	8.224	0.166	0.747	0.536	8.294

**Table 5:** Top five important variables identified by GBDT and SAPS II.

Ranks	GBTD	SAPS II
1	Pao2/Fio2 ratio	Glasgow Coma Score
2	Glasgow Coma Score	Age
3	Serum urea nitrogen level	Chronic diseases
4	Systolic blood pressure	Systolic blood pressure
5	White blood cell count	White blood cell count

## Data Availability

Data supporting reported results in this study can be found at https://eicu-crd.mit.edu/ (accessed on 20 December 2018) and https://mimic.mit.edu/ (accessed on 20 December 2018).
